# Effects of coenzyme Q10 supplementation on lipid profiles and liver enzymes of nonalcoholic fatty liver disease (NAFLD) patients: A systematic review and meta‐analysis of randomized controlled trials

**DOI:** 10.1002/fsn3.3315

**Published:** 2023-03-13

**Authors:** Ali Ardekani, Reza Tabrizi, Elham Maleki, Kamran Bagheri Lankarani, Seyed Taghi Heydari, Mehdi Moradinazar, Maryam Akbari

**Affiliations:** ^1^ Health Policy Research Center Institute of Health, Shiraz University of Medical Sciences Shiraz Iran; ^2^ Noncommunicable Diseases Research Center Fasa University of Medical Sciences Fasa Iran; ^3^ Clinical Research Development Unit, Valiasr Hospital Fasa University of Medical Sciences Fasa Iran; ^4^ Endocrinology and Metabolism Research Center, Institute of Basic and Clinical Physiology Sciences Kerman University of Medical Sciences Kerman Iran; ^5^ Research Center for Environmental Determinants of Health (RCEDH) Kermanshah University of Medical Sciences Kermanshah Iran

**Keywords:** antioxidants, coenzyme Q10, fatty liver, hyperlipidemias, liver function tests, non‐alcoholic fatty liver disease

## Abstract

As an antioxidant, coenzyme Q 10 (CoQ10) has been proposed as a possible treatment for non‐alcoholic fatty liver disease (NAFLD). In the present meta‐analysis, we aimed to determine the effects of CoQ10 supplementation on lipid profiles and liver enzymes of NAFLD patients. We searched PubMed, Web of Science, Scopus, and Cochrane Library on 21 April 2022 to retrieve randomized controlled trials on NAFLD patients in which CoQ10 was utilized as a treatment. Data were pooled using the random‐effects model and weighted mean difference (WMD) was considered as the summary effect size. The analysis of the six included studies indicated an overall non‐significant decrease in the lipid profiles (total cholesterol (TC), low‐density lipoprotein cholesterol (LDL), high‐density lipoprotein cholesterol (HDL), and triglyceride (TG)), and liver enzymes (aspartate transaminase (AST), alanine transaminase (ALT), and gamma‐glutamyltransferase (GGT)) of NAFLD patients who received CoQ10. Sensitivity analysis using “leave‐one out” method showed a significant reduction in AST, and GGT after excluding certain studies. Also, subgroup analyses showed significant difference based on CoQ10 dose for TC, AST, and GGT, and also a significant decrease in AST based on the duration of the intervention. No publication bias was found between the studies. Although an overall non‐significant decrease was observed in lipid profiles and liver enzymes of NAFLD patients, the results of sensitivity and subgroup analyses showed significant effects of CoQ10 in certain conditions. Further RCTs should be done in light of our findings.

## Graphical abstract

In the present article, we investigated the effects of coenzyme Q10 supplementation on liver enzymes and lipid profiles of NAFLD patients.

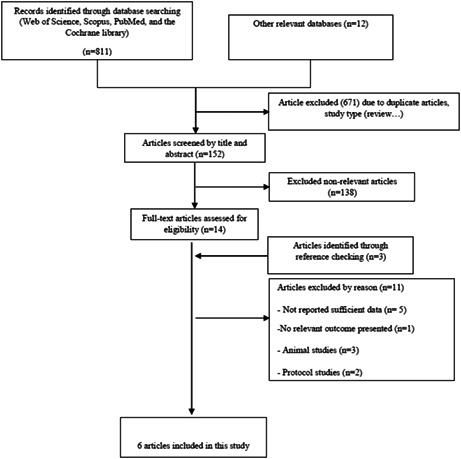

## 1 INTRODUCTION

The most frequent type of chronic liver disease is a non‐alcoholic fatty liver disease (NAFLD), which is notably prevalent in Western nations (Charlton, 2004; Masuoka & Chalasani, 2013). NAFLD encompasses an umbrella definition of a broad spectrum of clinical presentations, from simple uncomplicated steatosis to non‐alcoholic steatohepatitis (Farrell & Larter, 2006). During the past decades, alterations in human lifestyle have led to an increasing trend in obesity, high blood pressure, and type 2 diabetes mellitus (T2DM), all of which are risk factors for developing NAFLD (Godoy‐Matos et al., 2020; Zhao et al., 2020). According to a recent study, the worldwide prevalence of NAFLD from 1990 to 2019 increased from 21.9% to 37.3% (Le et al., 2021), which is notably high.

Although several meta‐analyses have indicated the potential benefits of a wide range of medicines and herbs such as green tea (Mansour‐Ghanaei et al., 2018), *Nigella sativa* (Tang et al., 2021), microbiome‐targeted therapies (Sharpton et al., 2019), Silymarin (Kalopitas et al., 2021), vitamin E (Amanullah et al., 2019), statins (Fatima et al., 2021), and some of the antidiabetic drugs (Lian & Fu, 2021), the treatment of NAFLD is still a matter of debate, and there are still no approved pharmacological treatments or prevention (Ferguson & Finck, 2021). Lifestyle modifications, weight loss through physical activity, and a healthy diet remain the core treatment of NAFLD (Cicero et al., 2018; Kenneally et al., 2017). Notably, antioxidants are suggested as a possible pharmacological treatment in previous studies (Mantovani & Dalbeni, 2021).

Coenzyme Q10 (CoQ10) is a lipid‐soluble antioxidant agent that may affect oxidative phosphorylation in mitochondria (Chokchaiwong et al., 2018; Testai et al., 2021). Previous investigations have revealed the possible role of CoQ10 in alleviating inflammation and cardiovascular diseases through its antioxidant effects (Hernández‐Camacho et al., 2018). Although poorly understood, it is hypothesized that CoQ10 could reduce lipid synthesis and help the oxidation of fatty acids through AMP‐activated protein kinase activation in the liver (Ke & Wenhua, 2019). Based on this hypothesis, studies were conducted to determine the possible therapeutic effects of CoQ10 in NAFLD patients, with mixed findings.

In the current investigation, we did a meta‐analysis on the randomized controlled trials (RCTs) of CoQ10 supplementation in NAFLD patients and investigated its effects on lipid profiles and liver enzymes after treatment.

## 2 METHODS

In conducting the present study, we utilized the “Preferred Reporting Items for Systematic Reviews and Meta‐Analyses” (PRISMA) statement (Page et al., 2021).

### 2.1 Search strategy

On 21st April 2022, we searched PubMed, Web of Science, Scopus, and Cochrane library databases using representative keywords and MeSH terms for “Coenzyme Q10” and “Non‐alcoholic fatty liver disease”. We searched Google Scholar to enhance our search's sensitivity. A forward and backward reference checking was also done for the included studies.

### 2.2 Screening process

We included RCTs on NAFLD patients (regardless of NAFLD grades) in which CoQ10 was used at least as a component of medications in the treatment group. No exclusion was posed based on year, language, or country of publication. Two independent authors evaluated the title and abstract of the studies after duplicate removal. Then, the remaining papers' full texts were evaluated based on the inclusion and exclusion criteria described above. Through contact with a third party, any disparities between authors were addressed throughout the screening process.

### 2.3 Data extraction and quality appraisal

The following data were extracted by two independent authors: first author's name, country, publication year, features of the study population, characteristics of intervention, and control groups before and after trial, including total cholesterol (TC), low‐density lipoprotein cholesterol (LDL), high‐density lipoprotein cholesterol (HDL), triglyceride (TG), aspartate transaminase (AST), alanine transaminase (ALT), and gamma‐glutamyltransferase (GGT). In addition, the quality of the studies was evaluated by two authors utilizing Cochrane Collaboration Risk of Bias tool (Higgins et al., 2011). In case of disagreements in the process of data extraction and quality appraisal, consultation with a third author was put forth.

### 2.4 Meta‐analysis

The effect sizes of CoQ10 supplementation on the changes of lipid profiles and liver enzymes were estimated. Effect sizes were assessed by using weighted mean difference (WMD) and its 95% CI and then pooling with random‐effects model.

In each article that did not report SD change, the following formula was applied to calculate the SD changes: “√[(SD_pre_^2 + SD_post_^2)–(2 × *r* × SD_pre_ × SD_post_)]”. The r value as a correlation coefficient was estimated at 0.8 using the following formula from included studies that reported the SD change: “*r* = [(SD_pre_^2 + SD_post_^2−SD_change_^2)/(2 × SD_pre_ × SD_post_)]”. If merely standard error (SE) was given, SE was converted to SD by the formula: “SD = SE × √n”.

Inter‐study heterogeneity was evaluated using the Chi‐squared test (with *p*‐value <.1) and *I* ^2^ statistic (*I* ^2^ value >50%). Sensitivity analysis was done using the “leave one out” method. Also, subgroup analyses were utilized to detect the source of heterogeneity. Egger's regression and Begg's rank correlation tests were statistically used to assess the evidence of small‐study effects among included trials. STATA version 16.0 (Stata Corp., College Station, TX) and Comprehensive Meta‐analysis (CMA) Version 2.0 were used for all statistical analyses.

## 3 RESULTS

### 3.1 Literature search and quality appraisal

**FIGURE 1:**
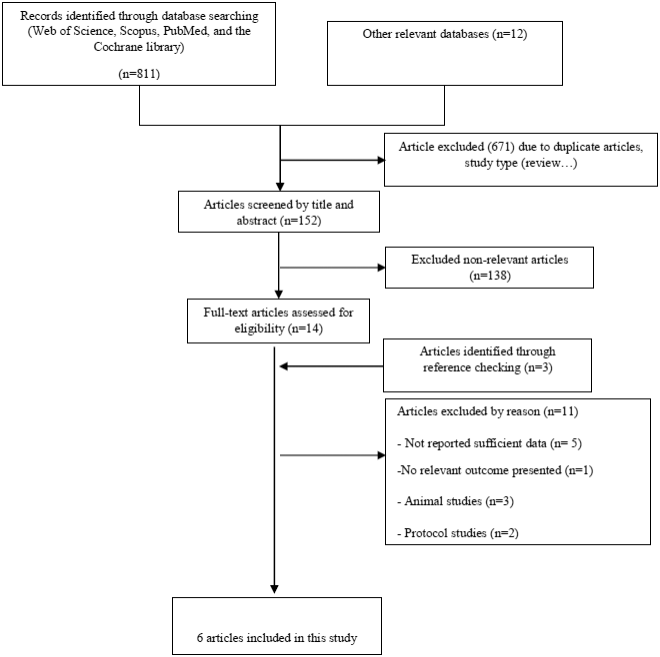
Screening and selection flowchart.

**FIGURE 2:**
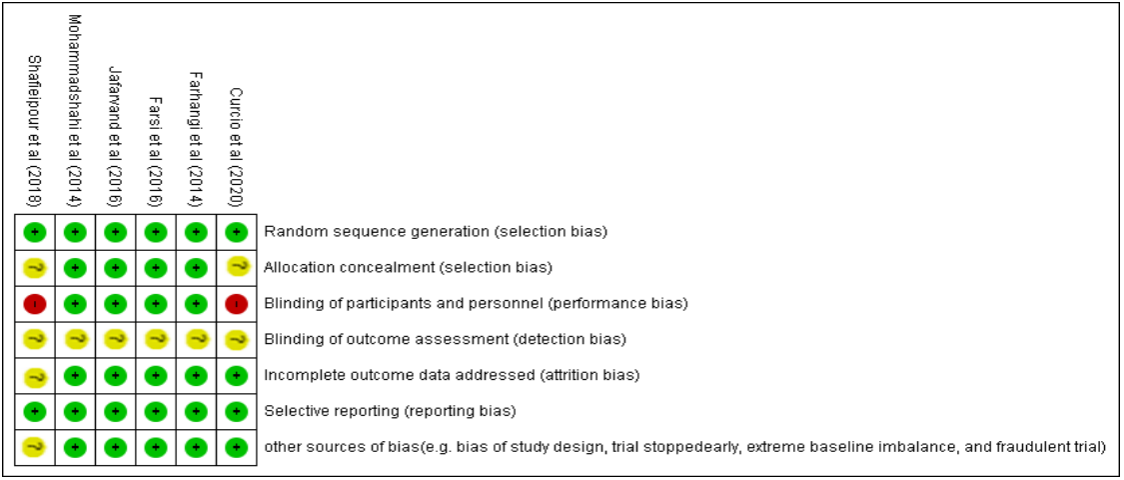
Details of risk of bias assessment.

Searching through the databases, we found 823 studies. Finally, after duplicate removal and the screening process, six studies (Curcio et al., 2020; Farhangi et al., 2014; Farsi et al., 2016; Jafarvand et al., 2016; Mohammadshahi et al., 2014; Shafieipour et al., 2018) satisfied the criteria for inclusion in the meta‐analysis (Figure 1). The features of the included RCTs are represented in Table 1. The results of the risk of bias assessment are depicted in Figure 2.

**TABLE 1:** Summary of the included studies.

First author	Country	Population target	Type intervention	Type control	Duration of intervention	Intervention/control group
Farhangi et al. (2014)	Iran	NAFLD patients	100 mg oral CoQ10 daily	Placebo daily	4 weeks	20/21
Farsi et al. (2016)	Iran	NAFLD patients	100 mg oral CoQ10 daily	Placebo daily	12 weeks	20/21
Jafarvand et al. (2016)	Iran	NAFLD patients	100 mg oral CoQ10 daily	Placebo daily	4 weeks	20/21
Mohammadshahi et al. (2014)	Iran	NAFLD patients	100 mg oral CoQ10 daily	Placebo daily	12 weeks	20/21
Shafieipour et al. (2018)	Iran	NAFLD patients	60 mg CoQ10 daily + lifestyle modification	Vitamin E 800 IU daily + lifestyle modification	12 weeks	30/25
Curcio et al. (2020)	Italy	Mild to severe NAFLD patients	2 capsules a day containing silymarin, vitamin C, vitamin E, coenzyme Q10 (20 mg), and selenomethionine +recommendations for lifestyle modification	Only recommendations for lifestyle modification	12 weeks	41/40

### 3.2 The effects of CoQ10 on NAFLD

**FIGURE 3:**
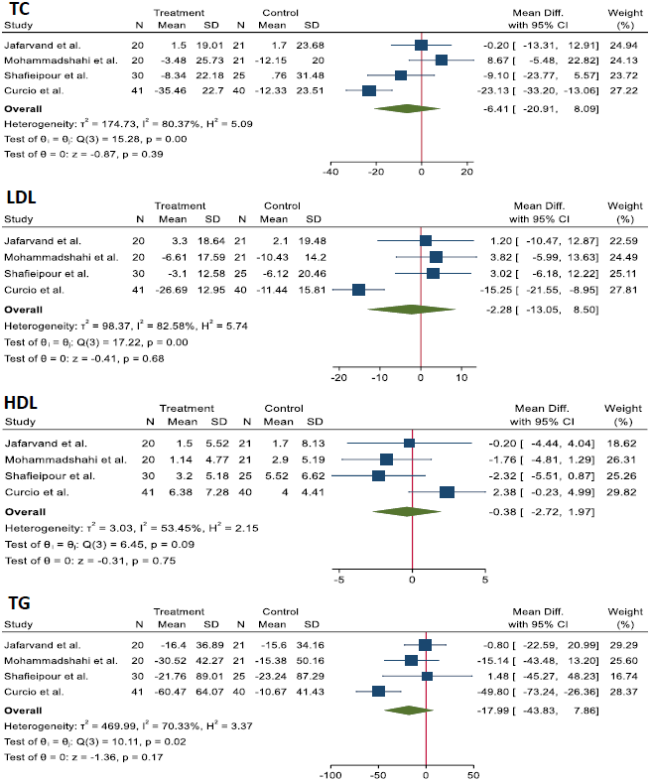
The effects of CoQ10 supplementation on lipid profiles of the NAFLD patients.

**FIGURE 4:**
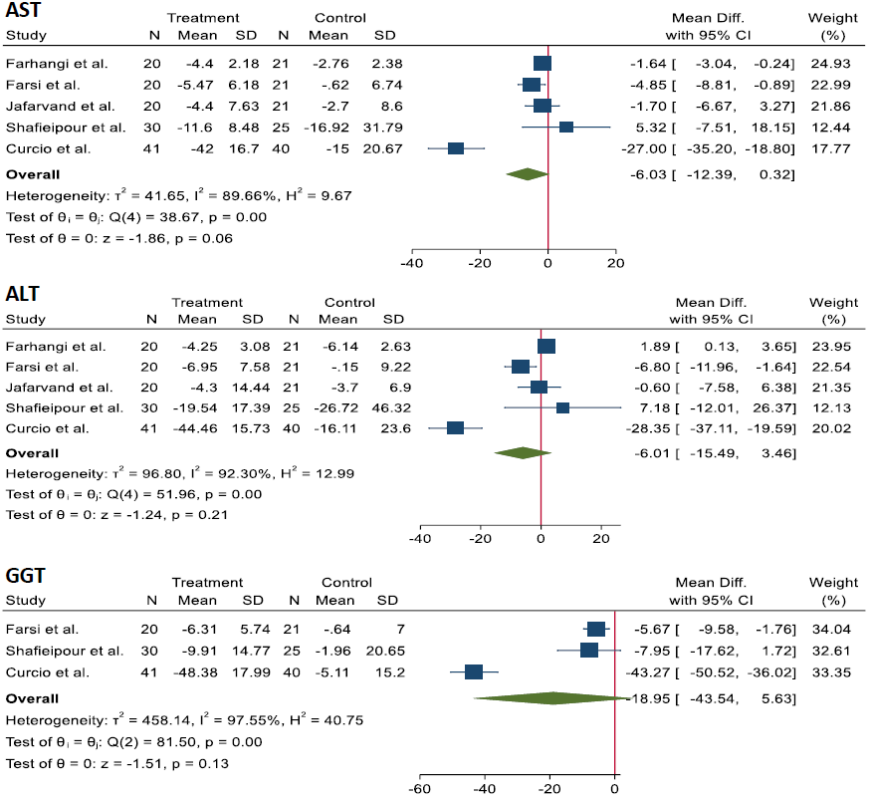
The effects of CoQ10 supplementation on liver enzymes of the NAFLD patients.

The effects of CoQ10 supplementation on lipid profiles and liver enzymes are shown in Figures 3 and 4. The analysis showed a non‐significant decrease in the lipid profile including TC [WMD = −6.4 mg/dL; 95% CI, −20.91; 8.09, *p* = .39; *I* ^2^ = 80.37%], LDL [WMD = −2.28 mg/dL; 95% CI, −13.05; 8.5, *p* = .68; *I* ^2^ = 82.58%], HDL [WMD = −0.38 mg/dL; 95% CI, −2.72; 1.97, *p* = .75; *I* ^2^ = 53.45%], and TG [WMD = −17.9 mg/dL; 95% CI, −43.83; 7.86, *p* = .17; *I* ^2^ = 70.33%] in the CoQ10 group compared to control group.

A non‐significant decrease was observed in the WMD of liver enzymes in the CoQ10 group in comparison with control group: AST [WMD = −6.03 U/L; 95% CI, −12.39; 0.32, *p* = .06; *I* ^2^ = 89.66%], ALT [WMD = −6.01 U/L; 95% CI, −15.49; 3.46, *p* = .21; *I* ^2^ = 92.3%], GGT [WMD = −18.95 U/L; 95% CI, −43.54; 5.63, *p* = .13; *I* ^2^ = 97.55%].

### 3.3 Subgroup analysis

In the subgroup analysis, we found significant effects of CoQ10 supplementation on lowering TC [WMD = −17.17 mg/dL; 95% CI, −30.76; −3.57, *p* = .013; *I* ^2^ = 58.13%] with <100 mg dose, and in “CoQ10 + other drugs” subgroups; however, this decrease was observed in AST [WMD = −2.12; 95% CI, −3.72; −0.52, *p* = .009; *I* ^2^ = 11.60%] and GGT [WMD = −5.67; 95% CI, −9.58; −1.76, *p* = .004] with ≥100 mg CoQ10 per day, and in “CoQ10 only” subgroups. Also, AST reduction [WMD = −1.64; 95% CI, −2.98; −0.30, *p* = .01; *I* ^2^ = 92.65%] was statistically significant in 4‐week intervention group (Table 2).

**TABLE 2:** Subgroup analyses for liver enzymes and lipid profiles.

Variables	Subgroup	Study	WMD (95% CI)	*p*	*I* ^2^ (%)
TC					
Duration	12 weeks	3	−8.33 (−27.3, 10.63)	.38	84.65
	4 weeks	1	−0.2 (−13.31, 12.91)	.97	–
Dosage	<100 mg	2	−17.17 (−30.76, −3.57)	**.013**	58.13
	≥100 mg	2	3.89 (−5.72, 13.51)	.427	0.0
Type of intervention	CoQ10 only	2	3.89 (−5.72, 13.51)	.427	0.0
	CoQ10 + other drugs	2	−17.17 (−30.76, −3.57)	**.013**	58.13
LDL					
Duration	12 weeks	3	−3.19 (−16.75, 10.35)	.64	87.32
	4 weeks	1	1.2 (−10.46, 12.86)	.84	–
Dosage	<100 mg	2	−6.43 (−24.32, 11.45)	.48	90.31
	≥100 mg	2	2.73 (−4.77, 10.24)	.47	0.0
Type of intervention	CoQ10 only	2	2.73 (−4.77, 10.24)	.47	0.0
	CoQ10 + other drugs	2	−6.43 (−24.32, 11.45)	.48	90.31
HDL					
Duration	12 weeks	3	−0.45 (−3.51, 2.59)	.76	68.97
	4 weeks	1	−0.2 (−4.43, 4.03)	.92	–
Dosage	<100 mg	2	0.12 (−4.48, 4.72)	.95	79.96
	≥100 mg	2	−1.22 (−3.70, 1.24)	.33	0.0
Type of intervention	CoQ10 only	2	−1.22 (−3.70, 1.24)	.33	0.0
	CoQ10 + other drugs	2	0.12 (−4.48, 4.72)	.95	79.96
TG					
Duration	12 weeks	3	−25.25 (−55.35, 4.84)	.10	63.82
	4 weeks	1	−0.8 (−22.59, 20.99)	.94	–
Dosage	<100 mg	2	−28.31 (−77.90, 21.27)	.26	72.93
	≥100 mg	2	−6.12 (−23.40, 11.14)	.48	0.0
Type of intervention	CoQ10 only	2	−6.12 (−23.40, 11.14)	.26	0.0
	CoQ10 + other drugs	2	−28.31 (−77.90, 21.27)	.48	72.93
AST					
Duration	12 weeks	3	−9.26 (−25.85, 7.33)	.27	92.65
	4 weeks	2	−1.64 (−2.98, −0.30)	**.01**	0.0
Dosage	<100 mg	2	−11.23 (−42.89, 20.43)	.48	94.23
	≥100 mg	3	−2.12 (−3.72, −0.52)	**.009**	11.60
Type of intervention	CoQ10 only	3	−2.12 (−3.72, −0.52)	**.009**	11.60
	CoQ10 + other drugs	2	−11.23 (−42.89, 20.43)	.48	94.23
ALT					
Duration	12 weeks	3	−10.72 (−28.47, 7.02)	.23	90.5
	4 weeks	2	1.74 (0.03, 3.44)	.04	0.0
Dosage	<100 mg	2	−11.65 (−46.40, 23.10)	.51	90.82
	≥100 mg	3	−1.55 (−7.34, 4.22)	.59	79.93
Type of intervention	CoQ10 only	3	−1.55 (−7.34, 4.22)	.59	79.93
	CoQ10 + other drugs	2	−11.65 (−46.40, 23.10)	.51	90.82
GGT					
Duration	12 weeks	3	−18.95 (−43.53, 5.63)	.13	97.55
	4 weeks	–	–	–	–
Dosage	<100 mg	2	−25.76 (−60.37, 8.85)	.14	96.95
	≥100 mg	1	−5.67 (−9.58, −1.76)	**.004**	–
Type of intervention	CoQ10 only	1	−5.67 (−9.58, −1.76)	**.004**	–
	CoQ10 + other drugs	2	−25.76 (−60.37, 8.85)	.14	96.95

*Note*: Bold values are statistically significant reduction.

### 3.4 Sensitivity analysis and publication bias

According to the sensitivity analysis, for AST after removing the study by Shafieipour et al. (WMD = −7.67; 95% CI, −14.57; −0.78) or Curcio et al. (WMD = −2.06; 95% CI, −3.83; −0.29), the results became significant. For GGT, after removing the study by Curcio et al. (WMD; −5.99; 95% CI, −9.61; −2.36), the results showed a significant effect. No significant difference was observed after removing each study from the pooled analysis for TC, LDL, HDL, TG, and ALT (File S1). Moreover, no publication bias was observed according to Egger's regression and Begg's rank correlation tests. Funnel plots of publication bias are available in File S2.

## 4 DISCUSSION

This is the first meta‐analysis on the effects of CoQ10 supplementation on NAFLD patients. In the present study, a statistically non‐significant decrease was observed in patients' AST, ALT, GGT, TC, LDL, HDL, and TG after treatment with CoQ10. However, according to the sensitivity and subgroup analyses, this reduction could be statistically significant in certain circumstances.

The hepatoprotective effects of CoQ10 have been reported in previous animal studies (Ashkani Esfahani et al., 2013; Choi et al., 2009; Jiménez‐Santos et al., 2014; Sohet et al., 2009), emphasizing the possibility of reduction in liver enzymes and inflammatory markers after treatment with CoQ10 (Perumpail et al., 2018). In line with their results, Farsi et al. (Farsi et al., 2016) by ultrasonographic evaluation of NAFLD patients before and after treatment found a significant improvement in the intervention group who received CoQ10. However, the mentioned study was the only study that investigated NAFLD grades after treatment. In our subgroup analysis, a significant reduction was observed in AST and GGT values of the patients who received at least 100 mg of CoQ10 per day. More studies are needed to evaluate both ultrasonographic grades and laboratory markers of NAFLD patients to determine the effects of CoQ10.

The interrelation between obesity and NAFLD has been suggested to be mediated by blood levels of lipids. Steatosis develops when the amount of liver fatty acid absorption from blood and de novo fatty acid synthesis exceeds their rate of oxidation and export (Fabbrini et al., 2010). In this regard, attenuated adiposity has been proposed as the mechanism by which CoQ10 may ameliorate NAFLD severity (Chen et al., 2019). Previous studies on animals indicated inconclusive outcomes of coenzyme Q supplementation on NAFLD (Botham et al., 2015). Similarly, in our meta‐analysis, a non‐significant decrease was observed among the patients who received CoQ10. However, given the paucity of studies, we strongly recommend further investigating CoQ10 supplementation in high‐quality RCTs with larger sample sizes.

Also, the duration of the intervention may be a key factor in determining the effectiveness of CoQ10; in an animal study that reported positive results, the course of the treatment was 24 weeks (Chen et al., 2019), while according to Table 2 in human studies, the duration of the treatment ranged from 4 to 12 weeks which yielded in mixed results. Further studies with longer intervention duration are recommended to clarify the impact of time.

According to the subgroup analysis, prescribing different dosages of CoQ10 may lead to different outcomes in NAFLD patients. CoQ10 has been utilized in a wide range of diseases like cardiovascular disorders, diabetes, Parkinson's disease, and mitochondrial cytopathies. The recommended dose of CoQ10 usually ranged from 50 mg to over 200 mg per day (Bonakdar & Guarneri, 2005). Comparative studies using different dosages of CoQ10 are needed to determine its lowering effects on liver enzymes and lipids. Also, as Crucio et al. (Curcio et al., 2020) utilized in their study, combination therapy of antioxidants, vitamins, and herbs, which has shown to be possibly effective in other studies, may be an effective strategy that should be further investigated in NAFLD patients.

We conducted the present meta‐analysis on RCTs with a standard protocol according to the PRISMA statement; however, it is not free of limitations. The paucity of studies and small sample sizes should be considered when interpreting the data. Also, most of the studies were from Iran, and one study was from Italy, which encompasses a narrow range of ethnicities. Furthermore, in one of the studies (Curcio et al., 2020), other minerals and vitamins were combined with CoQ10 in the intervention group, and in one study (Shafieipour et al., 2018) the control group received vitamin E.

## 5 CONCLUSION

Overall, a statistically non‐significant decrease was observed in lipid profiles and liver enzymes of NAFLD patients after treatment with CoQ10. However, the results of sensitivity and subgroup analyses indicated significant effects in certain circumstances. Further RCTs with large sample sizes using different dosages of CoQ10 in different intervention courses are required to establish a clearer picture of this topic.

## CONFLICT OF INTEREST STATEMENT

None.

## Supporting information


File S1.

File S2.

## Data Availability

The data supporting the current study are available upon reasonable request from the corresponding author.
