# Supplementary figures and images for: Effects of coenzyme Q10 supplementation on lipid profiles and liver enzymes of nonalcoholic fatty liver disease (NAFLD) patients: A systematic review and meta‐analysis of randomized controlled trials
Source: Food Sci Nutr. 2023 Mar 13;11(6):2580–8. doi: 10.1002/fsn3.3315 (PMC10261764; doi:10.1002/fsn3.3315)

**Supplementary file 1**. The results of sensitivity analysis.

**TC**

**LDL**


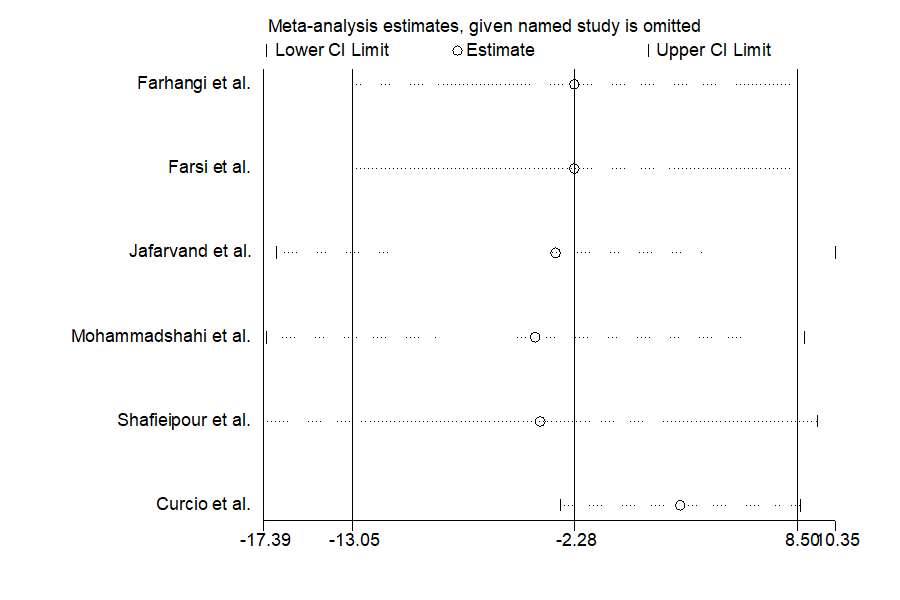


**HDL**


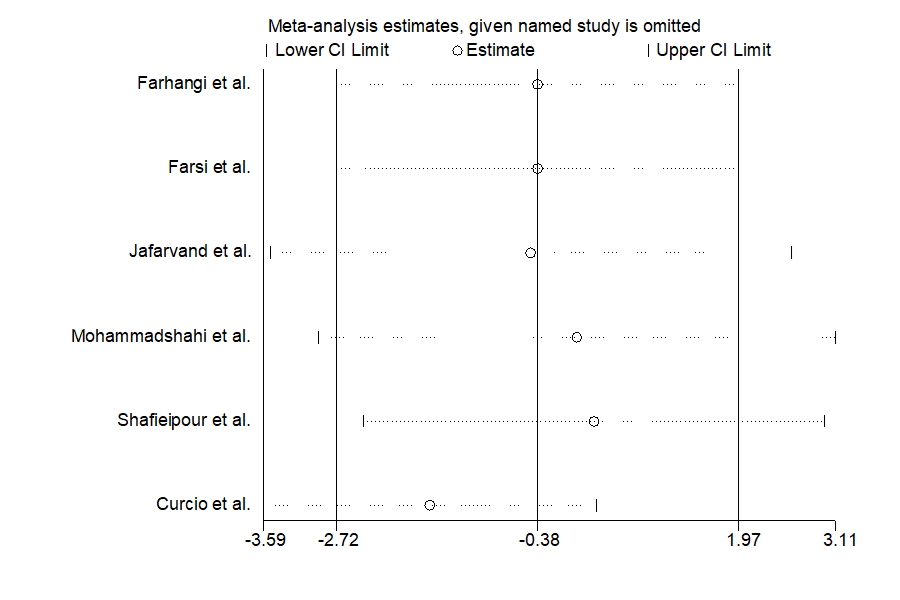


**TG**


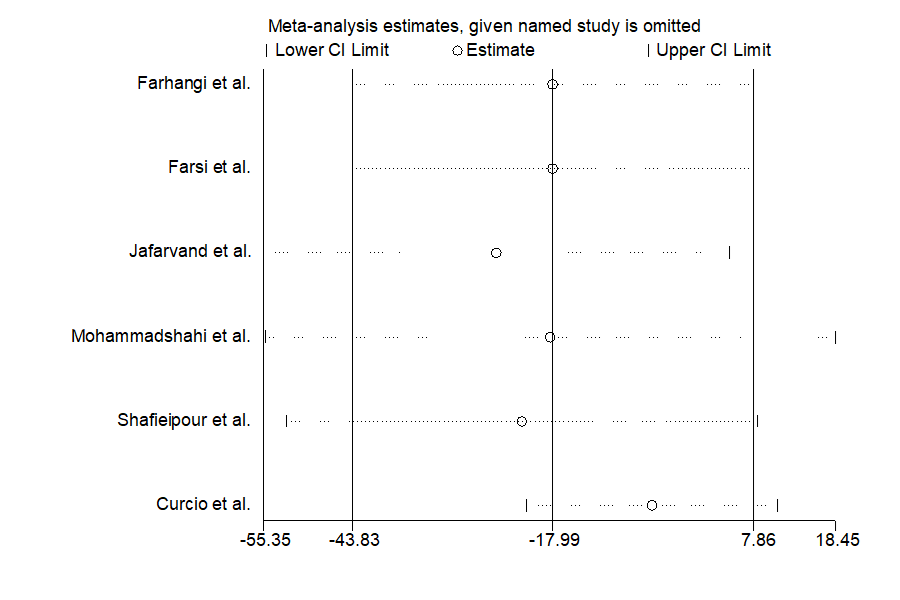


**AST**


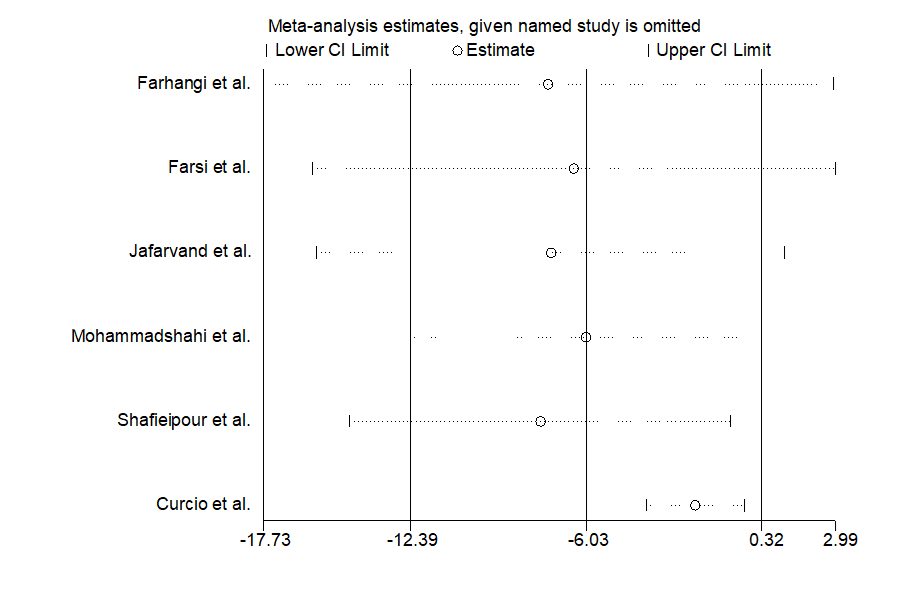


**ALT**

**
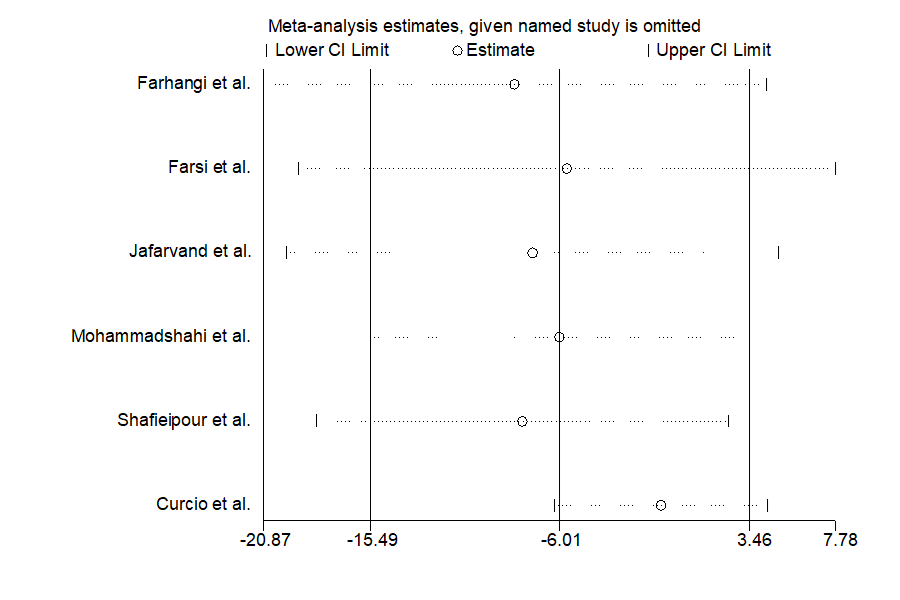
**

**GGT**

**
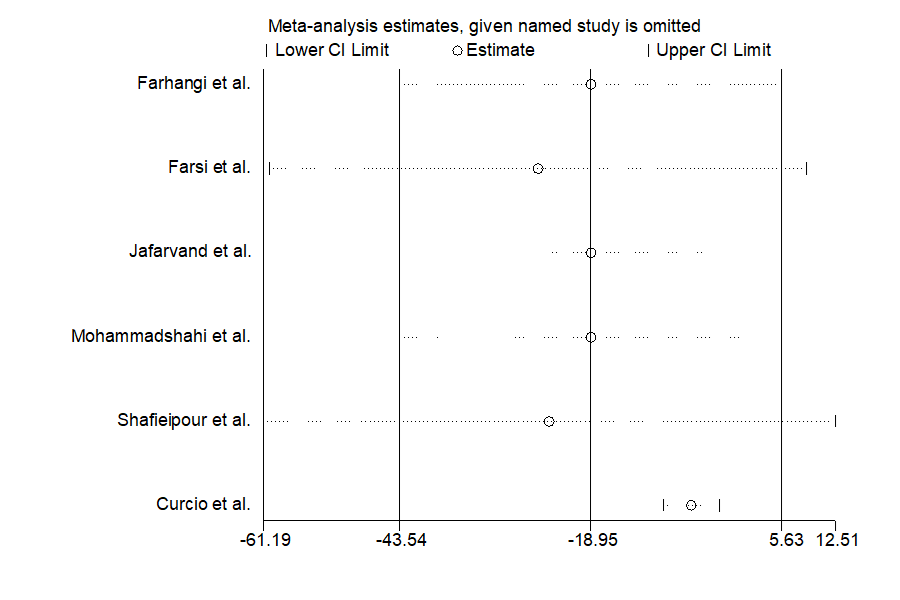
**

Supplement: Supplementary file 1 — File S1. [file FSN3-11-2580-s002.docx]
