# Supplementary material for: Effects of coenzyme Q10 supplementation on lipid profiles and liver enzymes of nonalcoholic fatty liver disease (NAFLD) patients: A systematic review and meta‐analysis of randomized controlled trials
Source: Food Sci Nutr. 2023 Mar 13;11(6):2580–8. doi: 10.1002/fsn3.3315 (PMC10261764; doi:10.1002/fsn3.3315)
Supplement: Supplementary file 2 — File S2. [file FSN3-11-2580-s001.docx]

**Supplementary file 2.** Funnel plots.

**TC**

**LDL**

**HDL**

**TG**

**AST**

**ALT**

**GGT**
